# Segmentation of Brain Tissues from Magnetic Resonance Images Using Adaptively Regularized Kernel-Based Fuzzy *C*-Means Clustering

**DOI:** 10.1155/2015/485495

**Published:** 2015-12-17

**Authors:** Ahmed Elazab, Changmiao Wang, Fucang Jia, Jianhuang Wu, Guanglin Li, Qingmao Hu

**Affiliations:** ^1^Shenzhen Institutes of Advanced Technology, Chinese Academy of Sciences, 1068 Xueyuan Boulevard, Shenzhen 518055, China; ^2^University of Chinese Academy of Sciences, 52 Sanlihe Road, Beijing 100864, China; ^3^Faculty of Computers and Information, Mansoura University, Elgomhouria Street, Mansoura 35516, Egypt

## Abstract

An adaptively regularized kernel-based fuzzy *C*-means clustering framework is proposed for segmentation of brain magnetic resonance images. The framework can be in the form of three algorithms for the local average grayscale being replaced by the grayscale of the average filter, median filter, and devised weighted images, respectively. The algorithms employ the heterogeneity of grayscales in the neighborhood and exploit this measure for local contextual information and replace the standard Euclidean distance with Gaussian radial basis kernel functions. The main advantages are adaptiveness to local context, enhanced robustness to preserve image details, independence of clustering parameters, and decreased computational costs. The algorithms have been validated against both synthetic and clinical magnetic resonance images with different types and levels of noises and compared with 6 recent soft clustering algorithms. Experimental results show that the proposed algorithms are superior in preserving image details and segmentation accuracy while maintaining a low computational complexity.

## 1. Introduction

Image segmentation is to partition an image into meaningful nonoverlapping regions with similar features. Segmentation of brain magnetic resonance (MR) images is necessary to differentiate white matter (WM), gray matter (GM), and cerebrospinal fluid (CSF). Such segmentation is essential for studying anatomical structure changes and brain quantification [1]. It is also a prerequisite for tumor growth modeling as tumors diffuse at different rates according to the surrounding tissues [2]. Due to potential existence of noise, bias field, and partial volume effect, segmentation of brain images remains challenging.

Image segmentation techniques can be roughly categorized into [3] thresholding, region growing, clustering, edge detection, and model-based methods. Clustering is an unsupervised learning strategy that groups similar patterns into clusters and can be hard or soft. Soft clustering is preferred as every pixel can be assigned to all clusters with different membership values [4, 5]. The most popular soft clustering methods applied to MR images are [4] fuzzy *C*-means (FCM) clustering [6, 7], mixture modeling, and hybrid methods of both.

Although the FCM algorithm comes with good accuracy in the absence of noise, it is sensitive to noise and other imaging artifacts. Therefore, enhancements have been tried to improve its performance by including local spatial and grayscale information [8–14] which will be briefly elaborated in Section 2.

A mixture model composed of a finite number of Gaussians has been employed for brain MR image segmentation. The main strategy to incorporate the local information into the mixture model is to use hidden Markov random fields for more accurate segmentation [15]. Nikou et al. [16] proposed a hierarchical and spatially constrained mixture model that takes into account spatial information by imposing distinct smoothness priors on the probabilities of each cluster and pixel neighborhoods. In [17], a nonparametric Bayesian model for tissue classification of brain MR images known as Dirichlet process mixture model was explored. Nguyen and Wu [18] introduced a way to incorporate spatial information between neighboring pixels into the Gaussian mixture model (GMM).

To be more robust to noise and attain fast convergence, FCM and GMM can be combined. Chatzis and Varvarigou [19] embedded the hidden Markov random field model into the FCM objective function to explore the spatial information. Chatzis [20] introduced a methodology for training finite mixture models under fuzzy clustering principle with a dissimilarity function to incorporate the explicit information into the fuzzy clustering procedure. Recently, Ji et al. [21] employed robust spatially constrained FCM (RSCFCM) algorithm for brain MR image segmentation by introducing a factor for the spatial direction based on the posterior probabilities and prior probabilities.

In [22], Li et al. presented an algorithm for brain tissue classification and bias estimation using a coherent local intensity clustering. Later they explored multiplicative intrinsic component optimization (MICO) [23] to improve the robustness and accuracy of tissue segmentation in the presence of high level bias field.

Generally, the current brain MR image segmentation algorithms suffer from one or more of the following shortcomings: lack of robustness to outliers [8, 9, 13], high computational cost [8, 13, 14, 16, 21], prior adjusting of crucial or many parameters [8–11, 21], limited segmentation accuracy in the presence of high level noise [8, 11, 19, 22, 23], and loss of such image details like CSF [9, 13, 14, 21]. In this paper, a new soft clustering framework is to be explored for better handling of the aforementioned segmentation problems.

The rest of this paper is organized as follows. Related work of FCM algorithm is presented in Section 2. The proposed framework is then elaborated in Section 3. Experiments on synthetic and clinical MR images are presented in Section 4. Sections 5 and 6 are devoted to discussion and conclusion, respectively.

## 2. Related Work

The FCM algorithm in its original form assigns a membership value to each pixel for all clusters in the image space. For an image *I* with set of grayscales *x*_*i*_ at pixel *i*  (*i* = 1, 2,…, *N*), *X* = {*x*_1_, *x*_2_, …, *x*_*N*_} ⊂ *R* ^*k*^ in *k*-dimensional space and cluster centers *v* = {*v*_1_, *v*_2_, …, *v*_*c*_} with *c* being a positive integer (2 < *c* ≪ *N*), there is a membership value *u*_*ij*_ for each pixel *i* in the *j*th cluster (*j* = 1,2,…, *c*). The objective function of the FCM algorithm is [7]

(1) $$\begin{array}{c} J_{FCM}=\overset{N}{\underset{i=1}{\sum }}\;\overset{c}{\underset{j=1}{\sum }}u_{ij}^{m}{\left\|\;x_{i}-v_{j}\;\right\|}^{2}, \end{array}$$

where *m* is a weighting exponent to the degree of fuzziness, that is, *m* > 1, and ‖*x*_*i*_ − *v*_*j*_‖^2^ is the grayscale Euclidean distance between pixel *i* and center *v*_*j*_. The membership *u*_*ij*_ should be constrained to the following:

(2) ∀i∈1,N,j∈1,c:∑j=1cuij=1,uij∈0,1,  0≤∑i=1Nuij≤N.

The membership function and cluster centers are updated iteratively in an alternating process known as alternate optimization. The membership function and cluster centers are

(3) uij=1∑k=1cxi−vj2/xi−vk21/m−1,vj=∑i=1Nuijmxi∑i=1Nuijm.

As the objective function in (1) does not include any local information, the original FCM is very sensitive to noise and the accuracy of clustering in the presence of noise and image artifacts will decrease. To overcome this problem, Ahmed et al. [8] modified the objective function by adding a term for the spatial information of neighboring pixels. This algorithm is denoted as FCM_S with the following objective function:

(4) JFCM_S=∑i=1N ∑j=1cuijmxi−vj2+αNR∑i=1N ∑j=1cuijm∑r∈Nixr−vj2,

where *α* is a parameter to control the spatial information of neighbors with 0 < *α* ≤ 1, *N*_*i*_ is the set of pixels around pixel *i*, and *N*_*R*_ is the cardinality of *N*_*i*_.

The FCM_S algorithm is computationally expensive as the local neighborhood term has to be calculated in each iteration step. To overcome this drawback, Chen and Zhang [10] replaced the term (1/*N*_*R*_)∑_*r*∈*N*_*i*__‖*x*_*r*_ − *v*_*j*_‖^2^ with ${\left\|{\overset{-}{x}}_{i}-v_{j}\right\|}^{2}$, where $\overset{-}{x}$ is the grayscale of a filtered image that could be calculated once in advance, and used kernel function to replace the Euclidean distance. The enhancement could be in two forms, that is, FCM_S1 by using the average filter and FCM_S2 by adopting the median filter. Their objective function is as follows:

(5) $$\begin{array}{c} J_{FCM\_S1,2}=\overset{N}{\underset{i=1}{\sum }}\;\overset{c}{\underset{j=1}{\sum }}u_{ij}^{m}{\left\|\;x_{i}-v_{j}\;\right\|}^{2}+\alpha \overset{N}{\underset{i=1}{\sum }}\;\overset{c}{\underset{j=1}{\sum }}u_{ij}^{m}{\left\|\;{\overset{-}{x}}_{i}-v_{j}\;\right\|}^{2}. \end{array}$$

Although the accuracy has been improved, it is sensitive to high level noises and different types of noises. In addition, the parameter *α*, which has a great impact on the performance, is set manually with care and requires prior information about noise.

Yang and Tsai [12] proposed a Gaussian kernel-based FCM method with the parameter *η*_*j*_ calculated in every iteration to replace *α* for every cluster. Similar to FCM_S1 and FCM_S2, this method has two forms: GKFCM1 and GKFCM2 for average and median filters, respectively. The parameter *η*_*j*_ is estimated using kernel functions:

(6) $$\begin{array}{c} \eta_{j}=\frac{\min_{j^{\prime }\neq j}\left(1-K\left(v_{\overset{´}{j}},v_{j}\right)\right)}{\max_{k}\left(1-K\left(v_{k},\overset{-}{x}\right)\right)}, \end{array}$$

where *K* is the kernel function. The replacement of *α* with *η*_*j*_ could yield better results than FCM_S1 and FCM_S2. However, for good estimation of *η*_*j*_, cluster centers should be well separated which might not be always true; hence the algorithm has to iterate many times to converge. Moreover, the learning scheme requires a large number of patterns and many cluster centers to find the optimal value for *η*_*j*_.

To tackle the problem of parameter adjustment, Krinidis and Chatzis [13] proposed the FLICM algorithm with a fuzzy factor that combined both spatial and grayscale information of the neighboring pixels. The fuzzy factor Gij=∑k∈Ni,i≠k1/1+dik1-uijmxi-vj2 was embedded into (1) as follows:

(7) $$\begin{array}{c} J_{FLICM}=\overset{N}{\underset{i=1}{\sum }}\;\overset{c}{\underset{j=1}{\sum }}\left[\;u_{ij}^{m}{\left\|\;x_{i}-v_{j}\;\right\|}^{2}+G_{ij}\;\right], \end{array}$$

where pixel *i* is the center of the local window, pixel *j* is in the neighborhood, and *d*_*ik*_ is the spatial Euclidean distance between pixels *i* and *k*.

Although FLICM algorithm enhances robustness to noise and artifacts, it is slow since the fuzzy factor (*G*_*ij*_) has to be calculated in each iteration. Moreover, *G*_*ij*_ is heavily affected by spatial Euclidean distance from the central pixel to its neighboring pixels to lose small image details due to the smoothing effect.

To enhance the FLICM algorithm, Gong et al. [14] developed KWFLICM algorithm with a trade-off weighted fuzzy factor to control the local neighbor relationship and replaced the Euclidean distance with kernel function. The weighted fuzzy factor ${\overset{´}{G}}_{ij}$ of KWFLICM is

(8) $$\begin{array}{c} {\overset{´}{G}}_{ij}=\sum_{k\in N_{i},i\neq k}\omega_{ik}{\left(\;1-u_{ij}\;\right)}^{m}\left(\;1-K\left(\;{x_{i},v}_{j}\;\right)\;\right), \end{array}$$

where *ω*_*ik*_ is the trade-off weighted fuzzy factor of pixel *k* in the local window around the central pixel *i* and 1 − *K*(*x*_*i*_, *v*_*j*_) is the kernel metric function. The trade-off weighted fuzzy factor combines both the local spatial and grayscale information [14]. Because of the trade-off weighted fuzzy factor, its computational cost increases substantially. In addition, the algorithm is unable to preserve small image details.

In addition to the abovementioned shortcomings, Szilágyi [24] pointed out serious theoretical mistakes in FLICM and KWFLICM. It was shown that the iterative optimization nature of FLICM and KWFLICM did not minimize their objective functions; instead, they iterated until the partition matrices converged. Furthermore, their objective functions intended to employ local contextual information but theoretically failed and were not even suitable for creating a valid partition [24].

To this end, a new way to modify the existing FCM clustering is explored with adaptive regularization for contextual information. The proposed framework utilizes a new parameter to control the effect of pixel neighbors based on the heterogeneity of local grayscale distribution. A weighted image is devised that combines the local contextual information with respect to the heterogeneity of local grayscale distribution and the original grayscale that is calculated once in advance to reduce the computational cost. To improve segmentation accuracy and robustness to outliers, a kernel function is employed to replace the Euclidean distance metric. Validation against both synthetic and clinical MR data has been carried out to compare the proposed algorithms with 6 recent soft clustering algorithms in terms of segmentation accuracy and computational costs.

## 3. Proposed Algorithms

We introduce a regularizing parameter to enhance segmentation robustness and preserve image details, devise a weighted image, and adopt the Gaussian radial basis function (GRBF) for better accuracy.

### 3.1. The Introduced Regularization Term

The parameter *α* used in [8–10] is usually set in advance to control the desirable amount of contextual information. Indeed, using a fixed *α* for every pixel is not appropriate since noise level differs from one window to another. In addition, setting such parameter needs prior knowledge about noise which is not always available in reality. Hence, adaptive calculation of *α* is necessary according to the pixel being processed.

To be adaptive to noise level of the pixel being processed, we first calculate the local variation coefficient (LVC) to estimate the discrepancy of grayscales in the local window to be normalized with respect to the local average grayscale. In the presence of noise to have high heterogeneity between the central pixel and its neighbors, LVC will increase. Consider

(9) $$\begin{array}{c} {LVC}_{i}=\frac{\sum_{k\in N_{i}}{\left(x_{k}-{\overset{-}{x}}_{i}\right)}^{2}}{N_{R}*{\left({\overset{-}{x}}_{i}\right)}^{2}}, \end{array}$$

where *x*_*k*_ is the grayscale of any pixel falling in the local window *N*_*i*_ around the pixel *i*, *N*_*R*_ is the cardinality of *N*_*i*_, and ${\overset{-}{x}}_{i}$ is its mean grayscale. Next, LVC_*i*_ is applied to an exponential function to derive the weights within the local window:

(10) ζi=exp⁡∑k∈Ni,i≠kLVCk,ωi=ζi∑k∈Niζk.

The ultimate weight assigned to every pixel is associated with the average grayscale of the local window:

(11) $$\begin{array}{c} \varphi_{i}=\left\{\begin{array}{cc} 2+\omega_{i}, & {\overset{-}{x}}_{i}<x_{i}\\ 2-\omega_{i}, & {\overset{-}{x}}_{i}>x_{i}\\ 0, & {\overset{-}{x}}_{i}=x_{i}. \end{array}\right. \end{array}$$

**Figure 1:**
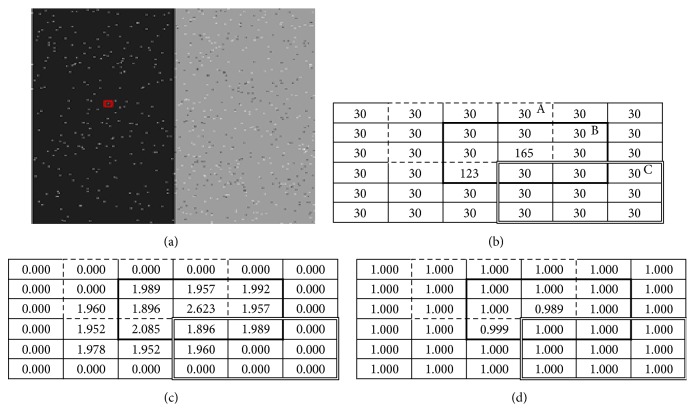
Calculation and effect of the regularization parameter in different cases. (a) Noisy image. (b) 6 × 6 subimage, red rectangle from (a), with 3 different windows A, B, and C. (c) Weights associated with each pixel using the proposed method. (d) Membership values after three iterations.

The parameter *φ*_*i*_ assigns higher values for those pixels with high LVC (for pixel *i* being brighter than the average grayscale of its neighbors, *φ*_*i*_ will be 2 + *ω*_*i*_, and *ω*_*i*_ will be large when the sum of LVC within its neighborhood is large) and lower values otherwise. When the local average grayscale is equal to the grayscale of the central pixel, *φ*_*i*_ will be zero and the algorithm will behave as the standard FCM algorithm. The value 2 in (11) is set through experiments to balance between the convergence rate and the capability to preserve details. The proposed parameter *φ*_*i*_ is embedded into (5) to replace *α*.  Figure 1 shows the calculation of *φ*_*i*_ with different cases of noise.

Here are some remarks on the parameter *φ*_*i*_.

The first point to emphasize is that *φ*_*i*_ is only relevant to the grayscales within a specified neighborhood, which is very different from FCM_S, FLICM, and KWFLICM, where the contextual information is expressed, respectively, by ∑_*x*_*r*_∈*N*_*i*__‖*x*_*r*_ − *v*_*j*_‖^2^, *G*_*ij*_, and ${\overset{´}{G}}_{ij}$, all containing a loop on the neighborhood and the cluster center *v*_*j*_. Due to its irrelevance to clustering parameters, *φ*_*i*_ could be calculated in advance before the clustering process which can greatly reduce the computational cost. On the contrary, FCM_S, FLICM, and KWFLICM will need to update the contextual weights at each iteration, which is the main reason why they have higher computational cost.

The second point is that the contextual information provided by *φ*_*i*_ is based on the heterogeneity of grayscale distribution within the local neighborhood, which is completely different from existing enhanced versions of FCM to base the contextual information on the difference between the grayscales of neighboring pixels and cluster centers. As a result, the proposed *φ*_*i*_ tends to yield a homogeneous clustering according to local grayscale distribution while existing enhanced FCM algorithms tend to make the clustering to have more homogeneous labels by incorporating the contextual information.

### 3.2. Devising a Weighted Image

In addition to making $\overset{-}{x}$, respectively, the grayscale of average/median filter of the original image, $\overset{-}{x}$ can also be replaced with the grayscale of the newly formed weighted image $\overset{-}{\xi }$:

(12) $$\begin{array}{c} {\overset{-}{\xi }}_{i}=\frac{1}{2+\max\left(\varphi_{i}\right)}\left(\;x_{i}+\frac{1+\max\left(\varphi_{i}\right)}{N_{R}-1}\underset{r\in N_{i}}{\sum }x_{r}\;\right), \end{array}$$

where *x*_*r*_ and *N*_*i*_ are, respectively, the grayscale and neighborhood of pixel *i* and *N*_*R*_ is the cardinality of *N*_*i*_. Formula (12) is inspired by the weighted image in [9] but utilizes *φ*_*i*_ explicitly to make the weighted image free from parameters that are difficult to adjust.

### 3.3. Measuring Distance Using Kernel Function

The Euclidean distance metric is generally simple and computationally inexpensive, but it is sensitive to perturbations and outliers. Recently, with popular usage of support vector machine, a new direction appears to use kernel functions. The kernel functions are able to project the data into higher dimensional space where the data could be more easily separated [25]. To do this, a so-called kernel trick has been adopted that can transform linear algorithm to nonlinear one using dot product [26]. Using the kernel trick, the Euclidean distance term ‖*x*_*i*_ − *v*_*j*_‖^2^ can be replaced with ‖*ϕ*(*x*_*i*_) − *ϕ*(*v*_*j*_)‖^2^ that is defined as

(13) ϕxi−ϕvj2=Kxi,xi+Kvj,vj−2Kxi,vj,

where *K* is the kernel function.

In this paper, we use GRBF kernel [27]:

(14) $$\begin{array}{c} K\left(\;{x_{i},v}_{j}\;\right)=\exp\left(\;-\frac{{\left\|x_{i}-v_{j}\right\|}^{2}}{2\sigma^{2}}\;\right), \end{array}$$

where *σ* is the kernel width.

Using GRBF, the kernel function in (13) will be

(15) $$\begin{array}{c} {\left\|\;\phi \left(\;x_{i}\;\right)-\phi \left(\;v_{j}\;\right)\;\right\|}^{2}=2\left(\;1-K\left(\;{x_{i},v}_{j}\;\right)\;\right). \end{array}$$

The choice of the kernel width *σ* is still a problem and has to be selected carefully. If it is large the exponential effect will be almost linear. On the contrary, if it is small, the cluster boundaries will be sensitive to outliers [27]. In [10], *σ* was set to a fixed value of 150 while in [12] the authors used sample variance to estimate *σ*. Similar to [14], we calculate *σ* based on the distance variances among all pixels:

(16) $$\begin{array}{c} \sigma ={\left[\;\frac{\sum_{i=1}^{N}{\left(d_{i}-\overset{-}{d}\right)}^{2}}{N-1}\;\right]}^{1/2}, \end{array}$$

where $d_{i}=\left\|x_{i}-\overset{-}{x}\right\|$ is distance from the grayscale of pixel *i* to the grayscale average of all pixels and $\overset{-}{d}$ is the average of all distances *d*_*i*_.

### 3.4. The Proposed Framework

The proposed adaptively regularized kernel-based FCM framework is denoted as ARKFCM. First, we calculate the adaptive regularization parameter *φ*_*i*_ associated with every pixel to control the contextual information using (11). The objective function is defined as

(17) JARKFCM=2∑i=1N ∑j=1cuijm1−Kxi,vj+∑i=1N ∑j=1cφiuijm1−Kx−i,vj.

Under the conditions specified in (2), the minimization of *J*_ARKFCM_(*u*, *v*) can be calculated through an alternate optimization procedure using (derivation is given in the Appendix)

(18) uij=1−Kxi,vj+φi1−Kx−i,vj−1/m−1∑k=1c1−Kxi,vk+φi1−Kx−i,vk−1/m−1

(19) vj=∑i=1NuijmKxi,vjxi+φiKx−i,vjx−i∑i=1NuijmKxi,vj+φiKx−i,vj.

When $\overset{-}{x}$ is replaced with the grayscale of the average/median filter of the original image, the algorithm is denoted as ARKFCM_1_/ARKFCM_2_. When ${\overset{-}{x}}_{i}$ is replaced with the weighted image ${\overset{-}{\xi }}_{i}$ defined in (12), the algorithm is denoted as ARKFCM_*w*_. The main steps for the proposed algorithms are as follows:

(1) Initialize threshold *ε* = 0.001, *m* = 2, loop counter *t* = 0, *v*, and *u* ^(0)^.
(2) Calculate the adaptive regularization parameter *φ*_*i*_.
(3) Calculate ${\overset{-}{x}}_{i}$ for ARKFCM_1_ and ARKFCM_2_ or $\overset{-}{\xi }$ for ARKFCM_*w*_.
(4) Calculate cluster centers *v*_*j*_^(*t*)^ using *u* ^(*t*)^ as in (19).
(5) Calculate the membership function *u* ^(*t*+1)^ with (18).
(6) If max ‖*u* ^(*t*+1)^ − *u* ^(*t*)^‖ < *ε* or *t* > 100 then stop; otherwise, update *t* = *t* + 1 and go to step (4).

## 4. Experiments

In this section, we present the experiments on both synthetic and clinical MR images. For validation, the proposed algorithms (ARKFCM_1_, ARKFCM_2_, and ARKFCM_*w*_) are compared with 6 recent soft clustering algorithms, namely, GKFCM1 [12], GKFCM2 [12], FLICM [13], KWFLICM [14], MICO [21], and RSCFCM [23]. As we are unable to have faithful implementation of RSCFCM [23], RSCFCM is compared only against the common data with results reported from [23]. The algorithms are implemented using MATLAB software package (a demo version is freely available online (http://www.mathworks.com/matlabcentral/fileexchange/54141-arkfcm-algorithm)). All the experiments are conducted with window size of 3 × 3 pixels, maximum number of iterations *t* = 100, and *ε* = 0.001. The accuracy of segmentation is measured using the Jaccard Similarity (JS) metric [28] which is defined as the ratio between the intersection and union of segmented volume *S*_1_ and ground truth volume *S*_2_:

(20) $$\begin{array}{c} JS\left(\;S_{1},S_{2}\;\right)=\frac{\left|S_{1}\cap S_{2}\right|}{\left|S_{1}\cup S_{2}\right|}. \end{array}$$

### 4.1. Experiments on Synthetic Brain MR Images

The following experiments are carried out using Simulated Brain Database (SBD) [29] which contains a set of realistic MR volumes produced by an MR imaging simulator with ground truths of CSF, GM, and WM available.

**Figure 2:**
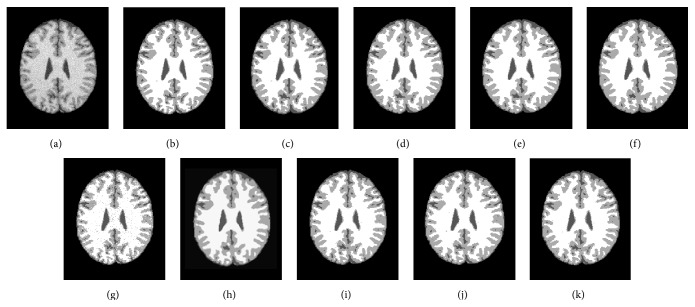
Segmentation results on a T1-weighted axial slice (number 100) from SBD with 7% noise and 20% grayscale nonuniformity. (a) Original image. (b) Ground truth. (c) GKFCM1 results. (d) GKFCM2 results. (e) FLICM results. (f) KWFLICM results. (g) MICO results. (h) RSCFCM results. (i) ARKFCM_1_ results. (j) ARKFCM_2_ results. (k) ARKFCM_*w*_ results.

The first experiment is to segment a T1-weighted axial slice (number 100) with 217 × 181 pixels corrupted with 7% noise and 20% grayscale nonuniformity into WM, GM, and CSF.  Figure 2 shows the segmentation results while Table 1 summarizes the JS and average running times.

**Figure 3:**
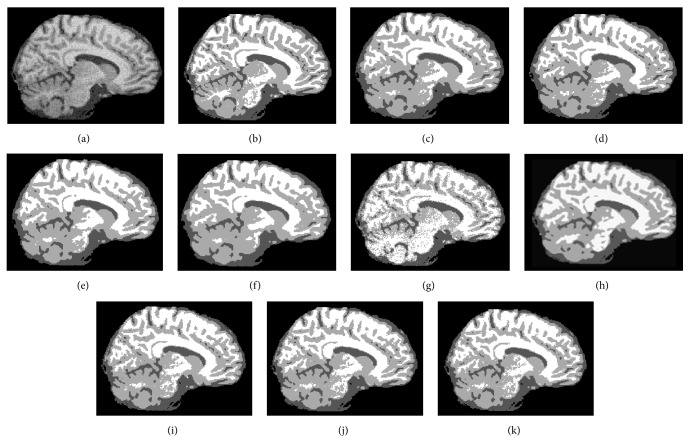
Segmentation results on a T1-weighted sagittal slice (number 100) from SBD with 7% noise and 20% grayscale nonuniformity. (a) Original image. (b) Ground truth. (c) GKFCM1 results. (d) GKFCM2 results. (e) FLICM results. (f) KWFLICM results. (g) MICO results. (h) RSCFCM results. (i) ARKFCM_1_ results. (j) ARKFCM_2_ results. (k) ARKFCM_*w*_ results.

The second experiment is to segment a T1-weighted sagittal slice (number 100) with 181 × 217 pixels corrupted with 7% noise and 20% grayscale nonuniformity. This image is chosen to show the capability of preserving details. The segmentation results and JS are presented in Figure 3 and Table 2, respectively.

**Figure 4:**
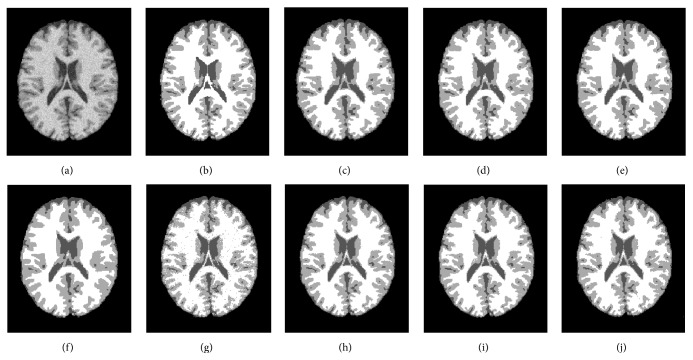
Segmentation results on a T1-weighted axial slice (number 91) from SBD with 10% Rician noise. (a) Original image. (b) Ground truth. (c) GKFCM1 results. (d) GKFCM2 results. (e) FLICM results. (f) KWFLICM results. (g) MICO results. (h) ARKFCM_1_ results. (i) ARKFCM_2_ results. (j) ARKFCM_*w*_ results.

The third experiment is to check the robustness to Rician noise that commonly affects MR images [30]. The segmentation results and the JS with average running times of a T1-weighted axial slice (number 91) with 217 × 181 pixels corrupted with 10% Rician noise are given in Figure 4 and Table 3, respectively.

### 4.2. Experiments on Clinical Brain MR Images with Tumors

**Figure 5:**
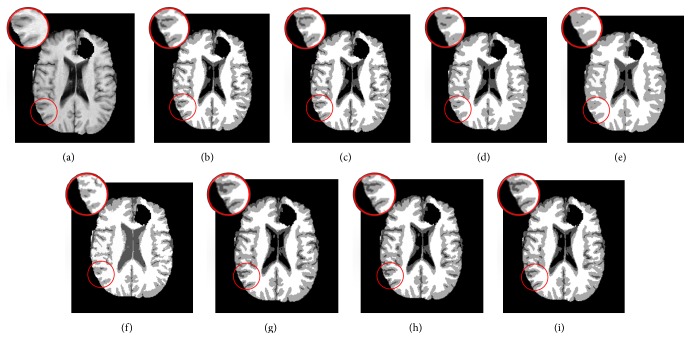
Segmentation results on the Brats1 image. (a) Original image. (b) GKFCM1 results. (c) GKFCM2 results. (d) FLICM results. (e) KWFLICM results. (f) MICO results. (g) ARKFCM_1_ results. (h) ARKFCM_2_ results. (i) ARKFCM_*w*_ results.

**Figure 6:**
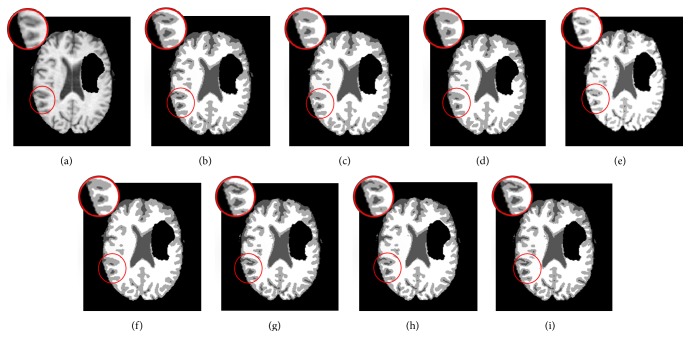
Segmentation results on the Brats2 image. (a) Original image. (b) GKFCM1 results. (c) GKFCM2 results. (d) FLICM results. (e) KWFLICM results. (f) MICO results. (g) ARKFCM_1_ results. (h) ARKFCM_2_ results. (i) ARKFCM_*w*_ results.

We experimented two T1-weighted axial slices (slices numbers 80 and 86, denoted, resp., as Brats1 and Brats2) with 240 × 240 pixels, respectively, from files pat266_1 and pat192_1 (available from MICCAI BRATS 2014 challenge, https://www.virtualskeleton.ch/BRATS/Start2014) (Figures 5 and 6). From black to white are, respectively, background, CSF, GM, and WM. It should be noted that clustering is carried out only for CSF, GM, and WM, with the pathology region being considered as background.

Since images Brats1 and Brats2 come only with ground truth for the pathology and have no ground truth for normal tissues, a quantification measure other than JS should be used. To this end, an entropy-based metric proposed by Zhang et al. [31] is adopted. This is to maximize the uniformity of pixels within each segmented region and to minimize the uniformity across the regions. First, the entropy of every region *j* is calculated:

(21) $$\begin{array}{c} H\left(\;R_{j}\;\right)=-\underset{m\in V_{j}}{\sum }\frac{L_{j}\left(m\right)}{S_{j}}\log\frac{L_{j}\left(m\right)}{S_{j}}, \end{array}$$

where *V*_*j*_ is the set of all possible grayscales in region *j*, *L*_*j*_(*m*) is the number of pixels belonging to region *j* with grayscale *m*, and *S*_*j*_ is the area of region *j*. For the grayscale image *I*, the *E* measure is calculated as

(22) $$\begin{array}{c} E=\overset{c}{\underset{j=1}{\sum }}\left(\;\frac{S_{j}}{S_{I}}\;\right)H\left(\;R_{j}\;\right)+\left(\;-\overset{c}{\underset{j=1}{\sum }}\left(\;\frac{S_{j}}{S_{I}}\;\right)\log\frac{S_{j}}{S_{I}}\;\right), \end{array}$$

where the first term represents the expected region entropy of the segmentation and the second term is the layout entropy. The measure *E* yields smaller value for better segmentation and higher otherwise.  Table 4 summarizes the segmentation accuracy in terms of *E* and running times for Brats1 and Brats2. It should be noted that the *E* metric is calculated only for WM, GM, and CSF regions without considering the background.

## 5. Discussion

FCM clustering is a well-known soft clustering method that assigns a membership degree for each pixel to every cluster. As the segmentation accuracy of FCM algorithm decays in the presence of noise, artifacts, and increased number of clusters, many investigations have been carried out on using contextual information to enhance the quality of segmentation. But how the contextual information shall be used effectively is still a challenge.

We propose an adaptively regularized kernel-based FCM clustering framework, with new parameter *φ*_*i*_ that adaptively controls the contextual information according to the heterogeneity of grayscale distribution within the local neighborhood. The new parameter is estimated using local variation coefficient among pixels within a specified neighborhood. A weighted image is devised that combines the original image and the parameter *φ*_*i*_ to represent the image contextual information embedded through the weighting procedure. Furthermore, a GRBF is adopted to replace Euclidean distance for better partitioning and to be less sensitive to outliers. The proposed framework can be in the form of 3 algorithms: ARKFCM_1_, ARKFCM_2_, and ARKFCM_*w*_ for the local average grayscale to be replaced with the grayscale of the average filter, median filter, and the devised weighted image, respectively. Experiments have been carefully carried out to show the superiority of the proposed algorithms in comparison with 6 recent soft clustering algorithms to be discussed further.

### 5.1. Segmentation Accuracy

The differences in JS between the proposed algorithms and the other 6 algorithms are clear for the axial slice with 7% noise and 20% grayscale nonuniformity (Table 1 and Figure 2) and become more distinctive in preserving details in the sagittal slice corrupted with the same noise (Table 2 and Figure 3). The average JSs of ARKFCM_1_, ARKFCM_2_, and ARKFCM_*w*_ are, respectively, 0.889, 0.892, and 0.891 for the axial slice as shown in Figure 2, which are better than the other 6 algorithms with range 1.3–4.8% (Table 1). For the sagittal slice with 7% noise and 20% grayscale nonuniformity shown in Figure 3, the average JSs of ARKFCM_1_, ARKFCM_2_, and ARKFCM_*w*_ are, respectively, 0.824, 0.825, and 0.825, which are better than the other algorithms with range 1.4–8.9% (Table 2). For the axial slice corrupted with 10% Rician noise shown in Figure 4, the average JSs of ARKFCM_1_, ARKFCM_2_, and ARKFCM_*w*_ are, respectively, 0.884, 0.886, and 0.881, which are better than the other algorithms with range 1.20–3.6% (Table 3). The higher JSs may imply that the proposed algorithms achieve a better balance in preserving image details in the presence of noise and grayscale inhomogeneity.

For the clinical brain MR images with tumors (Brats1 and Brats2) shown in Figures 5 and 6, the proposed algorithms achieve smaller *E* than other algorithms (Table 4). For Brats1/Brats2 image, ARKFCM_2_ attains the lowest *E* of 1.270/1.271, which is smaller than the other 5 algorithms with ranges 0.018–0.154 and 0.025–0.109, respectively. Visibly, GKFCM1 (Figures 5(b) and 6(b)) and GKFCM2 (Figures 5(c) and 6(c)) come with good results but they are unable to preserve small details such as CSF being wrongly broken possibly due to the difficulty in estimating the parameter *η*_*j*_. On the other hand, FLICM (Figures 5(d) and 6(d)) and KWFLICM (Figures 5(e) and 6(e)) yield smooth results and do not preserve many details which affects the segmentation accuracy of CSF (CSF is small as compared with surrounding GM and WM) due to the effects of *G*_*ij*_ and ${\overset{´}{G}}_{ij}$, respectively. For MICO algorithm (Figures 5(f) and 6(f)), the edges are not smooth enough; hence the CSF is not well preserved. Finally, results of ARKFCM_1_, ARKFCM_2_, and ARKFCM_*w*_ (Figures 5(g) and 6(g), Figures 5(h) and 6(h), and Figures 5(i) and 6(i)) show good balance between smooth borders and preserving image details due to the introduction of adaptive local contextual information measure *φ*_*i*_ to replace the fixed value of *α* or the oversmoothing factors *G*_*ij*_ or ${\overset{´}{G}}_{ij}$.

### 5.2. Computational Cost

In terms of computational cost, the objective function of FCM algorithm in its original form [6] contains only the difference between the grayscale of the current pixel *i* and the cluster centers *v*_*j*_. This is basically to cluster grayscales as there is no spatial information, so it has the smallest computational cost and can be implemented based on grayscale histogram to reduce the computational cost further [9, 11]. The enhancement of original FCM is to add the local contextual information to make it robust to noise and image artifacts at the expense of increased computational cost [7–14].

The GKFCM1 and GKFCM2 algorithms [12] use the parameter *η*_*j*_ to replace *α* and need an additional loop on the number of clusters to be calculated at each pixel to update the local contextual information. So they have higher computational cost than the original FCM, FCM_S1, and FCM_S2 at each iteration.

The FLICM algorithm [13] introduces *G*_*ij*_ which needs an additional loop on the neighborhood of the current pixel to calculate the local information in every iteration; thus it has a high computational cost. As an extension of FLICM, KWFLICM [14] introduces ${\overset{´}{G}}_{ij}$ that requires two additional loops on the neighborhood, so it has the highest computational cost at each iteration.

The RSCFCM algorithm [21] uses a spatial fuzzy factor that is constructed based on the posterior and prior probabilities and takes the spatial direction into account. That increases the complexity as many parameters have to be optimized and consequently the computational complexity.

MICO algorithm [23] comes with fast calculations due to the convexity of its energy function particularly in the presence of less noise but tends to have many iterations in the presence of high level noise.

The proposed algorithms incorporate the local contextual information by introducing LVC, which is a measure of grayscale heterogeneity and has nothing to do with the cluster centers. So it can be calculated once in advance and hence reduce the complexity of the clustering procedure.

**Figure 7:**
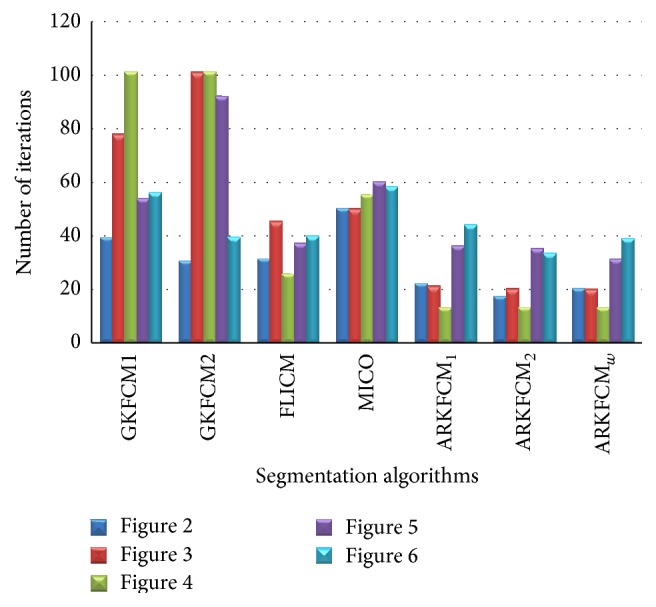
Average number of iterations of all the algorithms (except RSCFCM and KWFLICM) on Figures 2(a), 3(a), 4(a), 5(a), and 6(a).

The eventual computational cost will be the multiplication of the computational cost for each iteration and the number of iterations for convergence. The number of iterations will be dependent on the initialization as well as the objective function. To make fair comparisons, initializations are all set randomly and the average number of iterations for convergence is then recorded for 10 converged times. From Figure 7, it can be seen that the average iteration times will be data dependent, with ARKFCM_2_, ARKFCM_*w*_, and ARKFCM_1_ having the minimum number of iterations followed by FLICM, GKFCM2, and GKFCM1, respectively.

**Figure 8:**
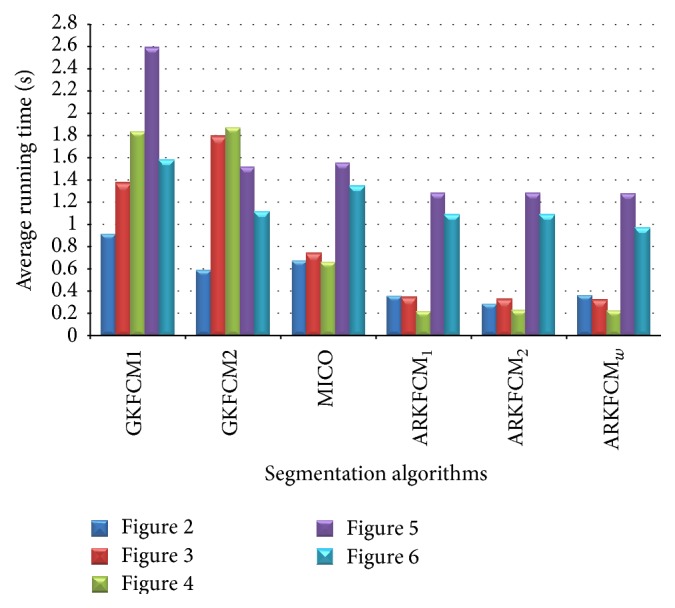
Average running times of all the algorithms (except FLICM, RSCFCM, and KWFLICM) on Figures 2(a), 3(a), 4(a), 5(a), and 6(a).

The running times for the tested images agree well with the above analysis as illustrated in Figure 8. For SBD images given in Figures 2, 3, and 4, the KWFLICM algorithm takes the longest time (124–166 seconds), followed by FLICM (3–5.4 seconds), RSCFCM (about 2.5 seconds), GKFCM1/GKFCM2 (0.6–1.9 seconds), and the proposed algorithms (0.22 to 0.36 seconds). For the clinical MR images in Figures 5 and 6, it takes more time (due to more iterations) as the images are more complicated, but the trend remains the same with the proposed algorithms having the lowest computational cost (Table 4 and Figure 8).

### 5.3. Neighborhood Size

The local neighborhood window size is a crucial factor to determine the smoothness of clustering and details to be preserved. We have experimented with different window sizes and found that a window size of 3 × 3 pixels achieves the best balance between segmentation accuracy and computational cost. Increasing window size to 5 × 5 pixels has very small impact on the JS but 7 × 7 pixels or more will, significantly, decrease the accuracy, such as losing image details like CSF. Therefore, it is recommended to use a local window of size 3 × 3 pixels for constructing the neighborhood.

### 5.4. Limitation

From the experiments, it was found that the proposed algorithms could be sensitive to severe noise in small areas between edges that have width 1 or 2 pixels (e.g., area between ventricles, Figures 4(h), 4(i), and 4(j)). Potentially, this may be solved by embedding edge detection technique into the clustering process which is yet to be explored.

## 6. Conclusion

An adaptively regularized kernel-based FCM framework has been proposed to enhance the original FCM for higher segmentation accuracy at low computational cost. The framework can be in the form of three algorithms that employ the heterogeneity of grayscales in the neighborhood employed for local contextual information. The main advantages are adaptiveness to local context, enhanced robustness, and independence of clustering parameters to decrease computational cost. GRBF kernel has been adopted as a distance metric. We validated the proposed algorithms on synthetic and clinical MR images with tumors. The proposed algorithms attain a higher JS (Tables 1, 2, and 3) and lower entropy measure *E* (Table 4) than the 6 recent soft clustering algorithms and could preserve small image details (Figures 2, 3, 4, 5, and 6). In addition, the proposed algorithms have a low computational cost and are, to the best of our knowledge, the only algorithms that are adaptive to local context and do not include cluster centers. Therefore, they attain a trade-off between high segmentation accuracy and low computational cost. The proposed algorithms can be a potential tool for segmenting brain MR images for further processing and other images as well.

## Figures and Tables

**Table 1:** JS and running time on the T1-weighted axial slice (number 100) from SBD with 7% noise and 20% grayscale nonuniformity.

Algorithm	GKFCM1	GKFCM2	FLICM	KWFLICM	MICO	RSCFCM	ARKFCM_1_	ARKFCM_2_	ARKFCM_*w*_
WM	0.930	0.933	0.941	0.937	0.894	0.898	0.940	0.941	0.940
GM	0.822	0.855	0.860	0.852	0.782	0.842	0.864	0.868	0.865
CSF	0.781	0.847	0.829	0.805	0.860	0.882	0.863	0.867	0.867
Average	0.844	0.879	0.876	0.865	0.845	0.874	**0.889**	**0.892**	**0.891**
Time (s)	0.911	0.586	3.403	139.470	0.673	2.530	**0.356**	**0.282**	**0.329**

**Table 2:** JS and running time on the T1-weighted sagittal slice (number 100) from SBD with 7% noise and 20% grayscale nonuniformity.

Algorithm	GKFCM1	GKFCM2	FLICM	KWFLICM	MICO	ARKFCM_1_	ARKFCM_2_	ARKFCM_*w*_
WM	0.773	0.775	0.771	0.765	0.672	0.785	0.788	0.786
GM	0.796	0.806	0.794	0.791	0.669	0.815	0.816	0.816
CSF	0.834	0.852	0.835	0.824	0.869	0.871	0.872	0.875
Average	0.801	0.811	0.800	0.794	0.736	**0.824**	**0.825**	**0.825**
Time (s)	1.377	1.797	5.392	166.717	0.751	**0.348**	**0.329**	**0.322**

**Table 3:** JS and running time on the T1-weighted axial slice (number 91) from SBD corrupted with 10% Rician noise.

Algorithm	GKFCM1	GKFCM2	FLICM	KWFLICM	MICO	ARKFCM_1_	ARKFCM_2_	ARKFCM_*w*_
WM	0.931	0.921	0.929	0.927	0.885	0.926	0.925	0.922
GM	0.804	0.827	0.831	0.821	0.777	0.838	0.840	0.834
CSF	0.826	0.872	0.861	0.846	0.889	0.887	0.892	0.887
Average	0.850	0.874	0.874	0.865	0.850	**0.884**	**0.886**	**0.881**
Time (s)	1.836	1.869	2.953	124.922	0.649	**0.218**	**0.220**	**0.218**

**Table 4:** Segmentation performance measure *E* and running time on the Brats1 and Brats2 images.

Image	Measure	GKFCM1	GKFCM2	FLICM	KWFLICM	MICO	ARKFCM_1_	ARKFCM_2_	ARKFCM_*w*_
Brats1	*E*	1.311	1.288	1.392	1.424	1.309	**1.274**	**1.270**	**1.279**
	Time (s)	2.591	1.516	9.510	635.413	1.747	**1.185**	**1.111**	**1.272**

Brats2	*E*	1.307	1.297	1.336	1.340	1.298	**1.273**	**1.271**	**1.279**
	Time (s)	1.576	1.115	5.170	400.295	1.546	**1.091**	**0.827**	**0.975**

## Appendix

The objective function *J*_ARKFCM_ given in equation (17) with conditions in (2) is a constrained minimization problem which can be solved using Lagrange multiplier method. Let the minimization problem of *J*_ARKFCM_ be written as follows:

(A.1) LARKFCMuij,vj,λi,φi=2∑i=1N ∑j=1cuijm1−Kxi,vj+2∑i=1N ∑j=1cφiuijm1−Kx−i,vj+∑i=1Nλi1−∑j=1cuij.

By taking the derivative of *L*_ARKFCM_ with respect to *u*_*ij*_ and setting the result to zero, for *m* > 1, we have

(A.2) ∂LARKFCM∂uij2muijm−11−Kxi,vj+2mφiuijm−11−Kx−i,vj−λi=0.

By using (A.2) we can calculate *u*_*ij*_ as follows:

(A.3) uij=λi2m1−Kxi,vj+φi1−Kx−i,vj1/m−1.

Since ∑_*k*=1_^*c*^ *u*_*ik*_ = 1  ∀*v*_*k*_, from (A.3) we can get

(A.4) ∑k=1cλi2m1−Kxi,vk+φi1−Kx−i,vk1/m−1=1,

(A.5) λi=1∑k=1c2m1−Kxi,vk+φi1−Kx−i,vk−1/m−1m−1.

By substituting (A.5) into (A.3) with necessary calculations, the final *u*_*ij*_ is

(A.6) uij=1−Kxi,vj+φi1−Kx−i,vj−1/m−1∑k=1c1−Kxi,vk+φi1−Kx−i,vk−1/m−1.

Similarly, by taking the derivative of *L*_ARKFCM_ with respect to *v*_*j*_ and setting the result to zero, we have

(A.7) ∂LARKFCM∂vj∑i=1NuijmKxi,vjxi−vj+∑i=1NφiuijmKx−i,vjx−i−vj=0.

After necessary calculations, the final *v*_*j*_ is

(A.8) $$\begin{array}{c} v_{j}=\frac{\sum_{i=1}^{N}u_{ij}^{m}\left(K\left(x_{i},v_{j}\right)x_{i}+\varphi_{i}K\left({\overset{-}{x}}_{i},v_{j}\right){\overset{-}{x}}_{i}\right)}{\sum_{i=1}^{N}u_{ij}^{m}\left(K\left(x_{i},v_{j}\right)+\varphi_{i}K\left({\overset{-}{x}}_{i},v_{j}\right)\right)}. \end{array}$$
